# Mixed Germ Cell Testicular Cancer with Left Ventricular Metastasis Presenting with Embolic Stroke and Small Bowel Tumor Seeding

**DOI:** 10.1155/2014/250531

**Published:** 2014-06-18

**Authors:** Srinath Sundararajan, Beth Braunhut, Frederick Ahmann, Amit Agarwal

**Affiliations:** ^1^Department of Hematology-Oncology, University of Arizona, Tucson, AZ 85724, USA; ^2^Department of Pathology, University of Arizona, Tucson, AZ 85724, USA

## Abstract

Testicular germ cell tumors (GCTs) metastasize in a very predictable fashion involving the retroperitoneal nodes first followed by hematogenous spread to distant organs like lungs, liver, and brain. Metastasis to heart is an extremely rare entity for GCT and fewer than 20 cases have been reported in the literature so far. We have summarized here a unique case of nonseminomatous germ cell tumor (NSGCT) with intracardiac metastasis resulting in systemic macroembolization to liver, spleen, brain, bowel and musculoskeletal tissues. This led to multiple adverse sequelae including ischemic stroke and bowel perforation.

## 1. Introduction

Testicular cancer is the commonest cancer in men between 20 and 40 years of age with a median age of diagnosis at 33 [1]. It had an estimated annual incidence of 7900 cases in 2013 and about 370 deaths [2]. Among these, germ cell tumors (GCTs) are the commonest type. Testicular cancers are generally believed to be chemotherapy sensitive tumors and have a high cure rate even in the metastatic setting [3]. Retroperitoneal lymph nodes and lungs are the commonest sites for metastasis for GCT and have better prognosis compared to other sites of metastasis [4]. Involvement of the heart in GCTs is very rare [5–7].

## 2. Case Report

29-year-old male with history of metastatic testicular cancer presented to the emergency department with sudden onset nausea, vomiting, seizure, and right-sided weakness for 2 days. A year ago, patient was diagnosed with metastatic nonseminomatous germ cell testicular cancer (NSGCT), mixed germ cell type with elevated beta human chorionic gonadotropin (beta HCG) and alpha fetoprotein (AFP) levels (Figure 4). Patient underwent orchiectomy, followed by 4 cycles of Bleomycin, Etoposide, and Cisplatin with partial response. He subsequently received 3 cycles of salvage chemotherapy with Etoposide, Ifosfamide, and Cisplatin. Two months prior to this admission, patient had metastasectomy of lung (Figures 2(c), 2(d), and 3), liver, and para-aortic lesions.

**Figure 1:**
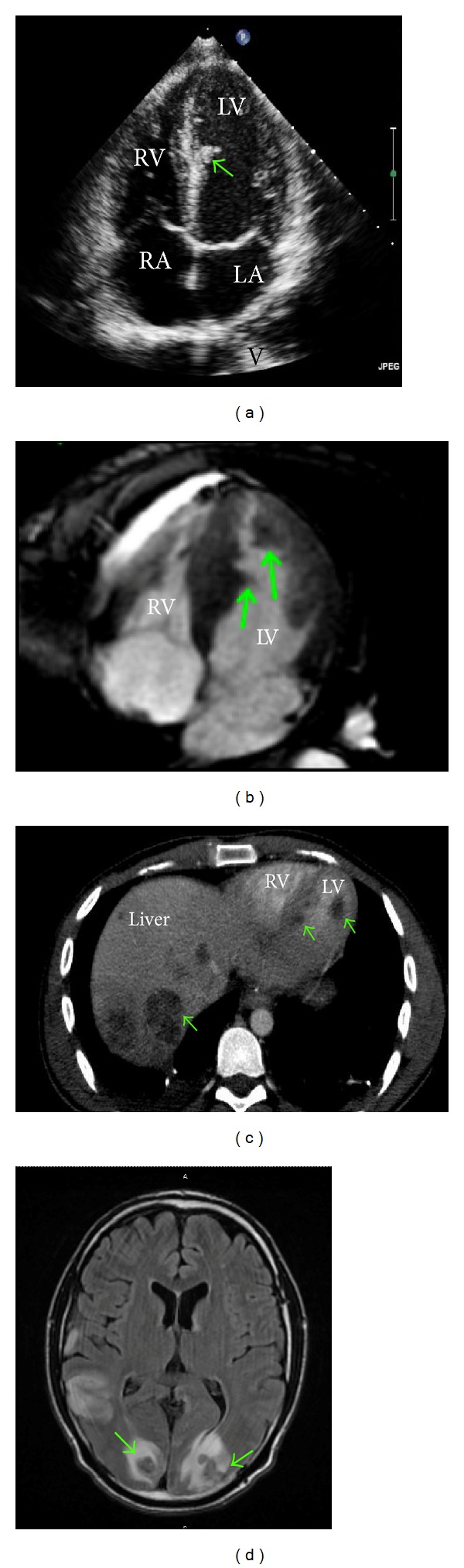
(a) An apical 4-chamber view of 2D echo showing mass in left ventricular cavity attached to left side of interventricular septum. (b) Cardiac MRI showing masses in left ventricular cavity attached to the interventricular septum and lateral wall. (c) CT chest and abdomen showing left ventricular masses and metastatic liver lesions. (d) MRI brain showing bilateral occipital lobe metastatic lesions surrounded by vasogenic edema.

On admission, his temperature was 98 Fahrenheit, pulse rate was 138, blood pressure was 106/21, and oxygen saturation was 99% in RA. Patient appeared clinically dehydrated and tachycardiac and had significant right-sided motor weakness with 0/5 muscle strength in the right upper and lower limbs. A MRI brain ordered to rule out stroke showed multifocal infarcts, numerous heterogeneous enhancing masses in bilateral frontal and occipital lobes consistent with brain metastases (Figure 1(d)). 2D echo (Figure 1(a)) showed an immobile mass attached to the left interventricular septum measuring 1 × 1 cm and multiple other small mobile left ventricular masses. Ejection fraction was 50–54% and bubble study was positive with minimal shunting from the right to the left during Valsalva maneuver. A cardiac MRI (Figure 1(b)) done following 2D echocardiography confirmed metastatic LV masses. Patient also was noted to have splenic and renal infarcts on further CT scans. A MRI of lower extremities ordered to evaluate an incidentally noted thigh mass showed metastatic lesions to skeletal muscles of bilateral thighs and gluteal region. Patient underwent whole brain radiation therapy for a total of 30 Gy in 10 fractions. After clinical improvement, patient was discharged home and was offered 3rd line chemotherapy.

**Figure 2:**
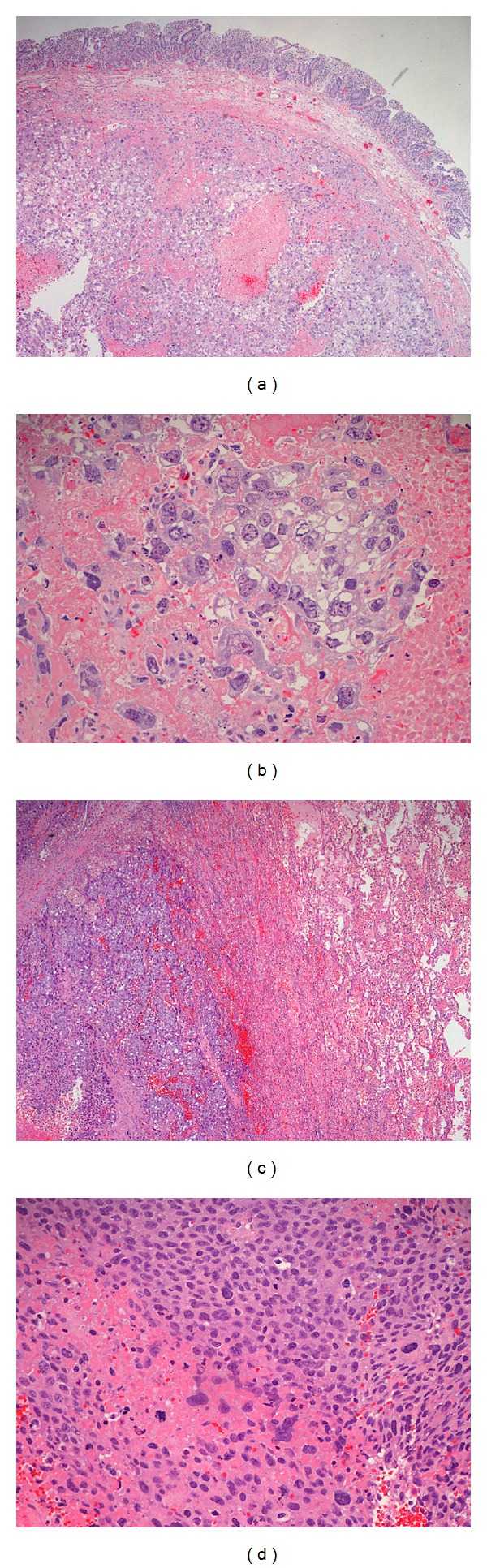
(a) Denuded small bowel mucosa overlying clusters of tumor cells with foci of hemorrhage and necrosis. (b) The tumor in small bowel composed predominantly of mononuclear cytotrophoblasts with clear cytoplasm, distinct cellular borders, and centrally located vesicular nuclei with prominent nucleoli. (c) Lung parenchyma (right) with adjacent nests of high-grade tumor cells (left) and hemorrhage. (d) Tumor cells from lung showing a mixture of mononuclear cytotrophoblasts and multinucleate syncytiotrophoblasts.

**Figure 3:**
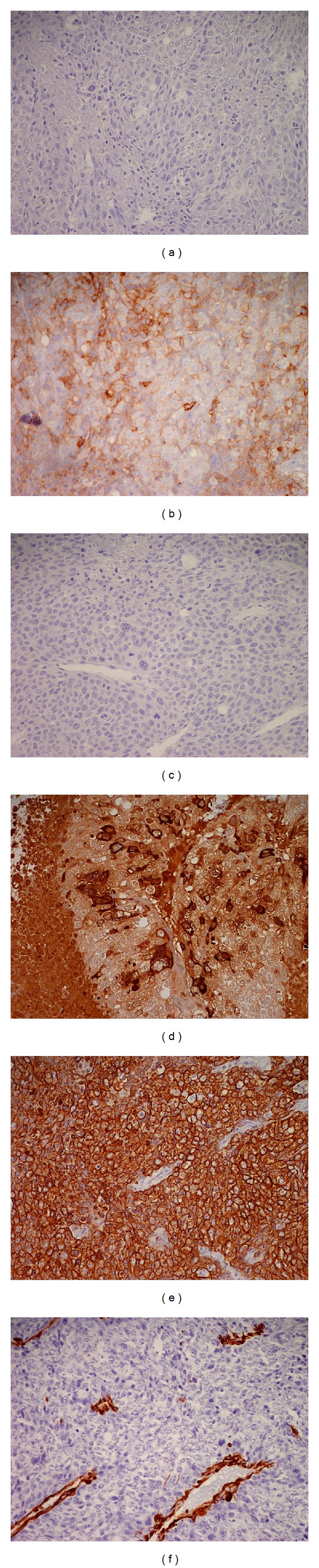
Immunohistochemistry from lung metastasis revealing choriocarcinoma with the tumor staining AFP negative (a), PLAP positive (b), CD30 negative (c), HCG positive (d), MAK 6 positive (e), and vimentin negative (f).

**Figure 4:**
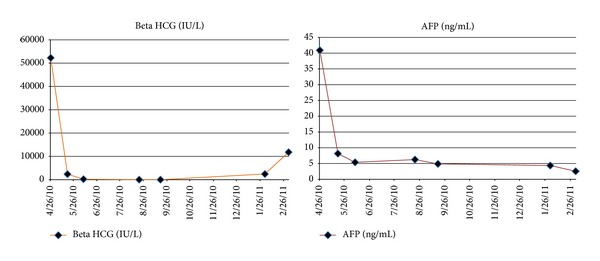
Trends of patient's beta HCG and AFP levels at diagnosis, during chemotherapy, and at relapse with cardiac, brain, liver, spleen, and skeletal muscle metastasis.

Within a few weeks, patient was readmitted to hospital for cycle 1 of Paclitaxel, Ifosfamide, and Cisplatin regimen. On day 6 of hospitalization, patient started having severe abdominal pain, distention, and hypotension. His labs revealed a neutropenia and lactic acidosis and plain X-ray abdomen showed free intraperitoneal air. Patient was evaluated by surgical team and was recommended to undergo an emergent laparotomy and small bowel resection. In the operation room, he was found to have intestinal perforation from tumor metastasis. Pathology showed extensive metastatic choriocarcinoma involving the muscular wall of small bowel (Figures 2(a) and 2(b)). His clinical status further worsened with respiratory failure and septic shock. His family resorted to comfort measures and he was categorized as do not intubate/do not resuscitate (DNI/DNR).

## 3. Discussion

Secondary cardiac tumors are not uncommon and were found incidentally in autopsy in about 8.4% patient with known diagnosis of a primary cancer of any site [8]. The most common primary tumors, which tend to metastasize to heart, are melanoma, lung, and breast cancers [9]. There are a few reported cases on testicular cancers metastasizing to heart in the literature. In a case series report of 19 cases, Weinberg et al. noted that the majority of metastatic testicular tumors involved the right side of the heart [5]. Direct hematogenous spread from inferior vena cava is believed to be the route of seeding [5, 10]. In the same report, it was noted that metastasis to left side of the heart especially left ventricle was extremely uncommon. Left ventricular metastasis could be due to variety of mechanisms including direct, hematogenous, and lymphatic spread [5, 9, 10]. In addition, in our patient, observed right to left shunt may have resulted in left ventricular tumor seeding. To our best knowledge, this is the second reported case of testicular cancer presenting with left ventricular metastatic lesions [5] and the first case with observed macroembolic phenomenon from left ventricular metastasis presenting with ischemic stroke and bowel perforation.

Cardiac metastasis can be pericardial, myocardial, or endocardial. Symptoms and clinical presentation of testicular tumor with cardiac metastasis can be varied. It can range from incidental murmur, shortness of breath, pleuritic chest pain, hemoptysis, or syncope to embolic phenomenon resulting in stroke and limb ischemia [5, 6, 10, 11].

Any patient with history of systemic malignancy presenting with the abovementioned symptoms should be evaluated with high index of suspicion for possible cardiac involvement. Transthoracic echocardiography is a good initial testing procedure for cardiac tumors with a diagnostic yield in the range of 90% [11]. Transesophageal echocardiography and cardiac MRI certainly provide additional advantage for smaller tumors with diagnostic yield reaching 100% [11, 12].

Left-sided cardiac tumors pose a significant risk of systemic embolization of tumor. In a retrospective study of 154 patients with primary or secondary cardiac tumors, Engberding et al. noted that systemic embolization was found in 11.9% of patients [11]. Common sites of embolization include cerebral arteries and retinal arteries although case reports of limb ischemia, pulmonary embolism, and renal infarcts are also noted in the literature [13–15].

Treatment of cardiac metastatic tumors is palliative and often depends on the nature of the underlying primary malignancy. Sensitive tumors like testicular germ cell tumors are treated with chemotherapy. However, tumors, which persist after chemotherapy, chemoresistant tumors like teratoma, or high-risk tumors may need surgical intervention in selected patients [16].

## 4. Conclusion

Testicular NSGCT are often diagnosed in early stages with limited disease. However, they can also present with metastatic disease at diagnosis like our patient. Heart, brain, and bowels are unusual sites of metastasis for testicular NSGCT and have worse outcomes. Cardiac metastasis from testicular cancers needs to be considered in a patient presenting with unexplained cardiac symptoms or stroke. Embolization of cardiac metastasis to other vital organs poses a great clinical challenge in managing such patients.
